# Niederlassungsprozesse von Hausärztinnen und Hausärzten in Bayern mit besonderem Fokus auf bürokratische Vorgänge

**DOI:** 10.1055/a-2637-3305

**Published:** 2025-07-18

**Authors:** Fabian Walter, Katharina Maier, Marco Roos, Klara Lorenz-Dant

**Affiliations:** 1 Allgemeinmedizin, Medizinische Fakultät Universität Augsburg, Augsburg, Germany

**Keywords:** Allgemeinmedizin, Niederlassung, Barrieren, Bürokratie, medical establishment, bureaucracy, barriers, general practice

## Abstract

**Hintergrund/Fragestellung:**

Der Bedarf an hausärztlicher Versorgung steigt bei sinkender Zahl an Hausärztinnen und Hausärzten. Wir untersuchten, wie Bürokratie im Niederlassungsprozess wahrgenommen wurde und sich die Wahrnehmung mit wachsender Niederlassungserfahrung verändert.

**Methodik:**

Mit Hilfe digitaler, leitfadengestützter Interviews wurden Ärztinnen und Ärzte mit Niederlassungswunsch bzw. im Niederlassungsprozess sowie, Ärztinnen und Ärzte in der Allgemeinmedizin mit bis zu 2 Jahren und 2 bis 5 Jahren Niederlassungserfahrung befragt. Die Aufzeichnungen wurden verbatim transkribiert und mit Hilfe der Thematic Analysis nach Braun & Clarke ausgewertet.

**Ergebnisse:**

Die 18 interviewten Personen identifizierten „Bürokratie“ in verschiedenen patientenfernen Bereichen. Mit Niederlassungserfahrung tritt ein Gewöhnungseffekt ein. Ressourcen zur Bewältigung der bürokratischen Herausforderungen wurden genannt.

**Schlussfolgerung:**

Die Teilnehmenden definieren Bürokratie als jede patientenferne Tätigkeit. Umgang mit diesen sollte trotz eintretender Gewöhnung erleichtert werden. Unterstützung durch Beratung oder elektronische Prozesse kann bei der Überwindung bürokratischer Hürden helfen und Positivbeispiele sollten systematisiert werden. Hausärztinnen und Hausärzte sehen Bürokratie als Hürde in der Niederlassung, sie beeinflusst aber nicht ihre Niederlassungsentscheidung. Der Eindruck von Bürokratie als wichtige Barriere in anderer Literatur wird dadurch relativiert.

## Abstract (en)

**Background:**

The demand for GP care is increasing while the number of GPs is decreasing. We investigated how GPs perceive bureaucracy during the process of establishing their own practice and how this perception changes with increasing establishment experience.

**Methodology:**

Physicians who wished to set up a practice or who were in the process of setting up a practice and those who, up to two years and two to five years previously, had set up a GP practice were interviewed digitally following an interview guide. The recordings were transcribed verbatim and analysed using Braun & Clarkes Thematic Analysis.

**Results:**

The 18 interviewees identified “bureaucracy” in various areas unrelated to patient care. A habituation effect occurs after the practice has been established. Resources for coping with bureaucratic challenges were mentioned.

**Conclusion:**

The participants define bureaucracy as any activity that is distant from the patient. Dealing with this should be made easier despite the habituation that occurs. Electronic processes and support through advice can help to overcome bureaucratic hurdles, and positive examples mentioned should be systematised. GPs view bureaucracy as a hurdle to setting up a practice, but it does not influence their decision to establish their own practice. Therefore, while still relevant, bureaucracy is found to be less important than previous research suggests.

## Einleitung

Durch den demographischen Wandel wächst einerseits der Bedarf an wohnortnaher, medizinischer Versorgung, andererseits fehlen zunehmend hausärztliche Leistungserbringer. Im Jahr 2024 gab es in Bayern 9.434 Hausärztinnen und Hausärzte mit einem durchschnittlichen Alter von 55 Jahren. Der Anteil der Personen über 60 Jahre war etwa 36% 1 . Die Gewinnung von hausärztlich Niedergelassenen hat daher eine hohe Relevanz.

Die hausärztliche Niederlassung in der vertragsärztlichen Versorgung von Patientinnen und Patienten in der gesetzlichen Krankenversicherung wird von den Kassenärztlichen Vereinigungen (KV) geregelt. Der Antrag auf Niederlassung, auch Zulassung zur vertragsärztlichen Versorgung, wird durch die jeweilige KV verwaltet. Die Zulassung wird dann von einem unabhängigen Gremium, dem jeder KV zugeordneten Zulassungsausschuss, erteilt.

Als Barrieren für eine Niederlassung werden in der Literatur Faktoren, wie die Vereinbarkeit von Familie und Beruf oder eine mangelnde finanzielle Unterstützung bei der Praxisgründung, beschrieben. Außerdem werden bürokratische Faktoren im Zulassungsprozess oder der Abrechnung genannt 2 3 4 . Bereits unter Medizinstudierenden werden „medizinfremde Tätigkeiten und Bürokratie“ als wichtige Barriere für eine Niederlassung gezählt 5 .

Mit dieser Studie sollte exploriert werden, wie Hausärztinnen und Hausärzte Bürokratie im Niederlassungsprozess oder der weiteren Niederlassung wahrnehmen und ob sich diese im Laufe der Niederlassung verändert.

## Methodik

Es wurden qualitative leitfadengestützte Interviews zur Exploration der subjektiven Wahrnehmungen und Definitionen von Bürokratie durchgeführt. Die Befragten waren Fachärztinnen und Fachärzte für Allgemeinmedizin (mit Niederlassungswunsch/im Niederlassungsprozess, mit bis zu zwei Jahren und bis zu 5 Jahren Niederlassungserfahrung). Dadurch sollten die Sichtweisen auf den Niederlassungsprozess zu verschiedenen Zeitpunkten dargestellt werden. Die Rekrutierung erfolgte über das Kompetenzzentrum Weiterbildung Allgemeinmedizin Bayern (KWAB). Es wurden alle Ärztinnen und Ärzte (n=34), die als Referierende 2023 im Kompetenzzentrum tätig waren, per E-Mail angeschrieben und zur Teilnahme eingeladen. Es erfolgte ein forschungspraktisches Sampling anhand einer positiven Zusage und der oben genannten Gruppenzugehörigkeit. Die Teilnehmenden wurden im Voraus über die Ziele der Studie informiert. Genaue Angaben über den Forschungshintergrund der Forschenden wurden nicht gemacht. Ein positives Ethikvotum der Ludwig-Maximilians-Universität München liegt vor (Referenz-Nummer 23-0229).

Zur Themeneingrenzung wurde eine Literaturrecherche zu Einflussfaktoren auf eine Niederlassungsentscheidung durchgeführt. Die hier aufgefundenen Themenbereiche wurden in einem Konsensustreffen zwischen KM, KL, FW und MR diskutiert, abgestimmt und zu einem Interviewleitfaden entwickelt. Für die Gruppen mit und ohne Niederlassungserfahrung wurden verschiedene Leitfäden entwickelt. Beide Leitfäden umschlossen sechs Themenbereiche. Wir eröffneten mit dem „beruflichen Weg in die Allgemeinmedizin und Niederlassungsentscheidung“, erfragten dann eine persönliche „Definition des Begriffs Bürokratie“ zur Einordnung der im Folgenden als bürokratisch gewerteten Hürden. Die weiteren Themen waren: „Erleben von Bürokratie im beruflichen Alltag“, „Erfahrungen mit Bürokratie in der Niederlassung“, „Erfahrungen mit Bürokratie im Niederlassungsprozess und (für die bereits niedergelassenen) Einordnung im Rückblick“ und „Vorschläge zur Reduzierung potenzieller Hürden“. Zuletzt wurden Verbesserungsvorschläge erbeten. Der Interviewleitfaden wurde in Probeinterviews mit ärztlichen Personen getestet.

Die Interviews wurden online über die Videoplattform ZOOM (Zoom Video Communications, Inc.) geführt und die Tonspur aufgezeichnet. Alle Teilnehmenden wurden einmalig interviewt. Die Interviews wurden jeweils in einem Tandem aus zwei Personen (KL/KM/FW) geführt und anschließend wortwörtlich transkribiert und anonymisiert. Die Transkripte wurden nicht zur Korrektur an die Teilnehmenden gegeben. Während der Interviews wurden Notizen angefertigt.

Die Auswertung erfolgte anhand der Thematic Analysis 6 . Die Analyse erfolgte zunächst unabhängig in zwei Gruppen. Einmal durch zwei wissenschaftliche Mitarbeitende (KL/FW) des Instituts für Allgemeinmedizin am Universitätsklinikum Augsburg (IAM) mit einem gesundheitswissenschaftlichen Hintergrund (KL: promoviert, FW: Masterabschluss) und einer medizinischen Doktorandin (KM) (niedergelassene Fachärztin für Allgemeinmedizin)

Um die Daten sowohl in der bestehenden Literatur zu verorten, als auch die Identifikation von anderen Aspekten zu ermöglichen, wurde ein deduktiv-induktives (KL/FW) und induktiv-deduktives (KM) Vorgehen gewählt. Die deduktiven Codes waren zuvor, basierend auf der oben genannten Literaturrecherche zu Einflussfaktoren auf die Niederlassung, erarbeitet worden. Zur Analyse wurde mit der Software Microsoft Word (Microsoft Corporation) gearbeitet.

Nach initialer Analyse der Daten diskutierten beide Gruppen die Zwischenergebnisse und konsentierten vier Kategorien: „Bürokratie im Niederlassungsprozess“, „Bürokratie im Praxisalltag“, „Unterstützung mit bürokratischen Prozessen“ sowie „Veränderung im Zeitverlauf“ (Online-Appendix 1). Insgesamt bestand eine hohe Übereinstimmung in den unabhängigen Codierungen. Datensättigung wurde diskutiert und festgestellt. Die Befragten wurden nicht um Feedback zu den Ergebnissen gebeten.

## Ergebnisse

### Beschreibung der Stichprobe

An den Interviews nahmen insgesamt n=18 Personen teil. 16 Ärztinnen und Ärzte aus dem kontaktierten Personenkreis nahmen nicht an der Studie teil. Die Befragungen fanden zwischen dem 10.05.2023 und dem 26.09.2023 statt und dauerten durchschnittlich 27,08 Minuten. Die demographischen Variablen sind in Tab. 1 zusammengefasst.

**Table:** **Tab. 1** Soziodemographische Variablen der Befragten

Alter	∅ 37 Jahre
	Range: 30 bis 52 Jahre
Geschlecht	Weiblich: 9 Personen
	Männlich: 9 Personen
Niederlassungserfahrung	Im Niederlassungsprozess befindlich oder Niederlassung geplant: 7 Personen
	bis 2 Jahre Erfahrung: 5 Personen
	2–5 Jahre Erfahrung: 6 Personen
Praxisart/Form der Niederlassung	Einzelpraxis: 6 Personen
	Gemeinschaftspraxis: 12 Personen
Gewünschte Form des Einstiegs, der noch nicht Niedergelassenen	Neugründung: 2 Personen
	Einstieg Gemeinschaftspraxis: 5 Personen
Form des Einstiegs der Niedergelassenen	Übernahme: 6 Personen
	Einstieg: 4 Personen
	Keine Angabe: 1 Person
Standort der Befragten	Land: 10 Personen
	Stadt: 6 Personen
	Keine Angabe: 2 Personen

### Der Niederlassungsprozess

#### Vertragsärztliche Zulassung

In der Mehrheit beschrieben die Befragten eine Redundanz von bürokratischen Prozessen rund um die vertragsärztliche Zulassung. So mussten wiederkehrend ähnliche Informationen bei Institutionen wie der KV, der Ärztekammer und dem Zulassungsausschuss angegeben werden. Zusätzlich wurde die Vergabe der vertragsärztlichen Zulassungen als intransparent eingeschätzt.

*„Was mich am meisten daran gestört hat, ist das die Formulare ja immer nach ähnlichen Dingen fragen, und man aber jedes Mal alles komplett wieder neu ausfüllen muss, weil nichts irgendwie digitalisiert hinterlegt ist, […]“*

Einige Befragte beschrieben den Zulassungsprozess als unproblematisch. Personen mit Niederlassungserfahrung berichteten eine „Gewöhnung“ an den Zulassungsprozess, im Zuge der Beantragung weiterer Zulassungen. Außerdem wurde davon berichtet, dass auch Sinn in den bürokratischen Verfahren gesehen wurde. Positiv bewertet wurden die unterstützenden Strukturen der Kassenärztlichen Vereinigung (z. B. Niederlassungsmentoring).

#### Praxisgründung/-übernahme

Manche Befragte fühlten sich nur unzureichend auf die Anforderungen weiterer bürokratischer Prozesse, wie Vertragsgestaltung, Finanzierung der Praxis oder benötigten Versicherungen vorbereitet. Der größte Bedarf an Unterstützung wurde in juristischen Themen gesehen.

*„Einfach die mangelnde Kenntnis an rechtlichen Dingen, also was muss alles in diesen Gesellschaftervertrag rein? Was bedeutet das dann, wenn es so formuliert ist in einem Juristendeutsch, weil ich weiß es einfach nicht. Ich kann das zehn Mal durchlesen aber letzten Endes ist es mir nicht klar was es bedeutet, und auch die Hürde quasi jemanden zu finden der einem das erklärt […]“*

Als unterstützende Ressourcen wurden Steuerberatungen oder Rechtsanwaltskanzleien wahrgenommen. Auch erfahrene Ärztinnen und Ärzte in zukünftigen Praxisgemeinschaften wurden als unterstützend betrachtet, allerdings mit der Einschränkung, dass diese gleichzeitig auch die Gegenparteien in Vertragsverhandlungen sein können. Insgesamt wurde zu kollegialem Austausch mit (externen) Kolleginnen und Kollegen geraten, die den Niederlassungsprozess bereits durchlaufen haben.

#### Unternehmerische Herausforderung/Verantwortung

Bisher unbekannte Aufgaben in der Praxisführung (Finanzrahmen und Steuern, Personalführung und Anforderungen an Informationstechnologie (IT)) wurden als bürokratische, zusätzliche zeitliche Belastung neben der Praxistätigkeit wahrgenommen. Die benötigte finanzielle Vorleistung (bis zur Honorierung der KV) für den geregelten Praxisbetrieb wurde als belastend empfunden.

*„Dazu kommt die Bürokratie im Sinne von Abrechnung, im Sinne von Personal, im Sinne von Qualitätsstandards, im Sinne von Hygiene, im Sinne von Sprechstundenbedarf bestellen, wie auch immer“*

Die Befragten mit Niederlassungserfahrung berichteten (in der Rückschau) von einer herausfordernden Summierung neuer Themen zum gleichen Zeitpunkt. Durch die wiederkehrende Auseinandersetzung wurde diese weniger herausfordernd.

Es wurden wichtige unterstützende Ressourcen für diese Themen benannt: sowohl privatwirtschaftliche Institutionen (z. B. Steuerberatungen), aber auch Niederlassungsberatung durch die KV oder die ärztlichen Interessenvertretungen (Hausärzteverband mit seinem Werkzeugkasten Niederlassung 1 und Fortbildungen oder kollegiales Feedback) werden hier genannt.

#### Insgesamt im Niederlassungsprozess wahrgenommene Unterstützung

Im Niederlassungsprozess erhielten die Befragten außerdem Unterstützung durch Eltern, Peers und Freunde mit Erfahrungswissen zur Niederlassung. Die Befragten wünschten sich mehr proaktive Unterstützung durch Institutionen, die an der Niederlassung beteiligt sind, wie beispielsweise der Kassenärztlichen Vereinigung (z. B. Beschreibung der Prozesse, Checklisten). Das Bilden eines Netzwerks im eigenen Fachbereich wurde befürwortet. Hilfsangebote wie der Werkzeugkasten Niederlassung oder der Erste Hilfe Kasten 2 wurden positiv wahrgenommen.

### Bürokratie im Praxisalltag

#### Telematikinfrastruktur

Die gesetzlichen Anforderungen zur Digitalisierung die durch die Telematikinfrastruktur umgesetzt werden, wird als Teil der Bürokratie wahrgenommen. Diese waren geprägt durch die hohe Anzahl an Prozessen in der Einrichtung und häufig benötigten Umstellungen (z. B. Beantragung des elektronischen Heilberufsausweises, Beschaffung von Konnektor und SMC-B-Karte, Einhaltung von Fristen). Auch wenn insgesamt positive Effekte zur verbesserten Kommunikation, Einsparung von Papier oder Arbeitserleichterung erhofft wurden, führten Systemausfälle und das System-Management zu zusätzlichen Belastungen.

*„Also Telematik, das empfinde ich als sehr Bürokratie lastig im Moment. Weil da einfach unheimlich viel Zeug zu erledigen ist, die ganze Beantragung von SMCB-Karte, Connector, sehe ich auch als Bürokratie im Moment ziemlich massiv.“*

Wenige Befragte schätzten die Telematikinfrastruktur als unproblematisch ein. Als Lösungen wurden die Delegation an Medizinische Fachangestellte und der Support durch IT-Firmen oder Hersteller von eingesetzter Software berichtet.

#### Abrechnung, Regresse und (befürchtete) juristische Konsequenzen

Die Anforderungen an die Genauigkeit bei Abrechnung und Dokumentation wurden als bürokratisch empfunden. Obwohl die Dokumentation als Möglichkeit zur Abwendung von Regressen angesehen wurde, empfanden die Befragten vor Regressen (das zeitversetzte Eintreten von finanziellen Belastungen in schwer zu antizipierender Höhe) eine hohe Furcht. Keine der befragten Personen hat eigene Erfahrungen mit Regressen gemacht.

*„Und bei den Ziffern, es gibt psychosomatische Ziffern, ähm, wo du eine bestimmte Dauer an Gesprächsführung theoretisch nachweisen musst. […]und wenn du dann aber nicht auf diese Gesamt ..ähm.. Menge von der Stundenzahl kommst, also was einfach unlogisch ist, weil du eigentlich mehr abgerechnet hast als der Tag Stunden hat, dann kriegst du natürlich Regress […]. […], die kommen halt immer, äh, Jahre später.“*

Personen mit höherer Erfahrung in der Niederlassung berichteten zu großen Teilen von einer Gewöhnung an die Prozesse. Jedoch wurden die häufigen Änderungen der Abrechnungsregelungen als belastend bewertet.

*„Also ich empfinde vor allem bürokratisch diese ganzen Emails von der KV kommen […] wo man wissen muss wenn jetzt irgendwelche Neuerungen sind und irgendwelche Ziffern, wie ich da abrechnen darf oder nicht […] irgendwelche Programme […] die dann aber nur für gewisse Krankenkassen gültig sind […]“*

Als positive Ressourcen wurden die Delegation von Teilen der Abrechnung an Medizinische Fachangestellte und die Unterstützung der KV (einjährige Abrechnungsberatung, Informationsmaterial) genannt.

#### Anfragen aus sozialmedizinischen Gründen

Anfragen aus sozialmedizinischen Gründen wurden als zeitlich intensive, bürokratische, rein ärztliche und dadurch nicht delegierbare Tätigkeit, wahrgenommen. Die Befragten berichteten von einer Zunahme der zu tätigenden Anfragen, welche meist mit dem Ausfüllen langer Formulare einhergingen. Genannt wurden Rehabilitations-Anträge (Reha-Anträge), Beantragung von Schwerbehindertenausweisen und Formulare zur Schul-/Arbeitsunfähigkeit. Solche Aussagen wurden vermehrt mit höherer Niederlassungserfahrung geäußert.

*„[…] also wahnsinnig nerven tun mich sozialmedizinische Fragen und Anfragen (lacht). Das nimmt einfach viel Raum und Zeit ist kompliziert, also das können sein irgendwelche Rentenfragen, Schwerbehindertengutachten, Reha-Anträge […]“*

#### Unternehmerische Verantwortung

Als weitere bürokratische Aspekte nannten die Befragten zeitaufwendige Aufgaben wie die IT-Verwaltung, den Datenschutz, die Finanz-Verwaltung, Hygiene- & Qualitätsmanagement und unvorhergesehene Verwaltungs-Tätigkeiten (bspw. nachträgliche Dokumentation von Corona-Tests).

*„Bürokratie ist das was mich im Moment jeden Tag in den Wahnsinn treibt und mir die Zeit raubt, die ich dann nicht mehr für meine Patienten und Patientinnen habe. […] ob es jetzt Dokumentation ist, ob es äh die Vereinbarungen von Terminen ist, ob es Qualitätsmanagement ist.. ähm.. oder irgendwelche Versorgungsamtanfragen, Versicherungen, den ganzen Papierkram den man dann in seiner Freizeit, am Wochenende irgendwann macht […]“*

Personen ohne Niederlassungserfahrung berichteten von Unsicherheiten in Bezug auf diese Aufgaben. Personen mit bis zu zwei Jahren Erfahrung berichteten von hilfreichen Erfahrungen aus der Anstellung und teilweise von Verständnis für die Prozesse. Manche Befragte mit bis zu fünf Jahren Niederlassungserfahrung betrachteten das Thema als zeitraubenden Störfaktor in der medizinischen Versorgung.

Zur Unterstützung delegierten die Befragten in diesem Bereich an Medizinische Fachanagestellte oder suchten Unterstützung durch die KV oder Steuerberater. Auch Eltern mit eigener hausärztlicher Niederlassungserfahrung stellten eine wichtige Ressource dar. Gewünscht wurden Weiterbildungen zu den genannten Thematiken.

## Diskussion

Die befragten Ärztinnen und Ärzte berichteten von bürokratischen Herausforderungen in verschiedenen Themengebieten. Generell wurde alles als bürokratisch angesehen, was nicht direkt mit ärztlicher Tätigkeit am Patienten verbunden war. Vor allem Aufgaben der Unternehmensgründung/-leitung wurden als bürokratisch bezeichnet. Befragte die bereits niedergelassen waren, berichteten von einer Gewöhnung an die bürokratischen Prozesse. Die bürokratischen Anteile des Niederlassungsprozess wurden im Nachhinein, als einmalig auftretende Vorkommnisse angesehen. Mit fortschreitender Niederlassungserfahrung wurde der Sinn der bürokratischen Vorgänge deutlicher. Für die Bewältigung der bürokratischen Aufgaben im Niederlassungsprozess und im Praxisalltag wurden unterschiedliche unterstützende Ressourcen benannt: externe Expertise auf arztfernen Gebieten (IT, Finanzen, Juristerei), kollegiale Unterstützung und die ärztliche Selbstverwaltung. Besonders die KV wurde in der Anfangsphase der Niederlassung als wichtige Unterstützung betrachtet. In den Aussagen wurde deutlich, dass es keine einheitliche oder effizienteste Vorgehensweise gibt. Vielmehr wurde am häufigsten eine Kombination aus Angeboten der ärztlichen Selbstverwaltung, Berufsverbänden, anderen Anbietern oder privaten sozialen Netzen angegeben. Die Gewichtung der verschiedenen Ressourcen blieb aber individuell.

Die Befragten definierten bürokratische Tätigkeiten breit und verstanden hierunter alles, was nicht unter die unmittelbaren medizinischen Tätigkeiten fiel. Die aufgefundenen Definitionen fanden sich auch in verschiedenen Studien zu Barrieren und Förderfaktoren zur Niederlassung in der Allgemeinmedizin, die einen wichtigen Einfluss von Bürokratie auf Berufszufriedenheit herstellten 3 7 .

Die Wahrnehmung vieler, berichteter bürokratischer Prozesse, verringerte sich mit zunehmender Niederlassungserfahrung. Primär zu nennen ist hier der einmalige Prozess der Niederlassung, aber auch wiederkehrende Prozesse wie beispielsweise die Abrechnung. Ähnlich wie von Ludwig et al. 8 dargelegt, zeigte sich ein Abbau der Barrieren durch die persönliche Erfahrung. Andere bürokratische Prozesse können systematisch verändert werden, da sie eine dauerhafte Belastung für die Befragten darstellten, wie beispielsweise ein überbordendes Formularwesen, vor allem im Bereich von sozialmedizinischen Anfragen, oder, die oben beschriebene, wiederholte erforderliche Übermittlung administrativer Informationen von den Gremien der ärztlichen Selbstverwaltung (gerade im Zulassungsprozess). Im Bürokratieindex 2019 wurde daher bereits eine Verschlankung des Zulassungsverfahrens (keine doppelten Vorlagen) vorgeschlagen 9 . Auch die digitale Arbeitsunfähigkeitsbescheinigung oder die Umsetzung des eRezepts wurden hier als sinnvoll eingeschätzt 9 . Auch im Bürokratieindex von 2022 wurde auf eine Arbeitserleichterung durch eine verbesserte Digitalisierung hingewiesen 10 .

Trotz dem politischen Willen zur Digitalisierung im Gesundheitswesen befindet sich Deutschland vor allem im internationalen Vergleich immer noch am Beginn des Prozesses 11 12 . Dies spiegelte sich in den als bürokratisch wahrgenommenen Anteilen der Telematikinfrastruktur. Es zeigte sich jedoch bei den Befragten, dass in der Digitalisierung auch positive Aspekte gesehen werden. Es wird daher vorgeschlagen, dass durch allgemeinmedizinische Praxiserfahrung die Digitalisierung des deutschen Gesundheitswesens mitgestalten sollte, um mögliche Barrieren abzubauen 13 .

Einige, als bürokratisch wahrgenommene, Prozesse sind der unternehmerischen Führung einer Praxis zuzuordnen. Auch in anderen Unternehmenszweigen werden bürokratische Vorgaben als hinderlich wahrgenommen 14 . Ärztinnen und Ärzte sind hier durch ihre fehlende Ausbildung in diesem Gebiet zusätzlich belastet. Inhalte dieser Art sind bisher nicht regelhaft in Curricula medizinischer Studiengänge verankert. Ebenso finden Sie bisher in der fachärztlichen Weiterbildung zu selten Raum. Hier gibt es erste Ansätze dem entgegenzuwirken: Mit Wahlpflichtfächern zur vertragsärztlichen Niederlassung an den medizinischen Fakultäten oder den, in den Interviews benannten, „Werkzeugkasten Niederlassung“ (Deutscher Hausärztinnen- und Hausärzteverband) und „Erste Hilfe Kasten“ (Kompetenzzentrum Weiterbildung Allgemeinmedizin Bayern). Damit wird individuelle kollegiale Beratung, wie sie in unserer Studie häufig genannt wurde, ergänzt und systemisch etabliert. Gleichzeitig sollte mehr Fokus auf die Vermittlung einer Haltung als Unternehmerin oder Unternehmer gelegt werden.

## Stärken und Schwächen

Eine Stärke ist die Fokussierung auf die individuelle Sichtweise junger Ärztinnen und Ärzte auf bürokratische Prozesse. Damit wurde entgegen bisher aus der Literatur bekannten Untersuchungen explizit auf den Einfluss von bürokratischen Prozessen auf die Niederlassung hin untersucht. Als Limitation muss angeführt werden, dass ausschließlich Personen befragt wurden, die sich im Niederlassungsprozess befinden oder bereits niedergelassen sind. Zudem wurden Ärztinnen und Ärzte eingeladen, die als Referierende im Kompetenzzentrum Weiterbildung Allgemeinmedizin tätig waren. Damit besteht eine positive Selektion. Ob Personen, die von einer Niederlassung absehen, den Hauptgrund in bürokratischen Prozessen sehen, sollte in weiteren Untersuchungen exploriert werden.

## Schlussfolgerung

In einer Gesamtbetrachtung zeigt sich, dass die Befragten bürokratische Prozesse zwar als Hürde wahrnehmen, aber dadurch nicht in ihrer Niederlassungsentscheidung beeinflusst werden. Viele der bürokratischen Prozesse wurden im Nachhinein als bewältigbar beschrieben und mit anderen wurde der Umgang erlernt. Damit ergänzt diese Befragung die bestehenden Kenntnisse von Barrieren und Motivatoren für eine Niederlassung und relativiert den Einfluss von „Bürokratie“ als eine gewichtige Barriere für eine vertragsärztliche Niederlassung junger Ärztinnen und Ärzte. Gleichzeitig kann durch einen Ausbau von Unterstützungsangeboten die wahrgenommene Belastung durch Bürokratie gesenkt werden.

## 

## Fundref Information

Beauftragter für Bürokratieabbau der Bayerischen Staatsregierung —

Bayerischer Normenkontrollrat —

## Footnotes

Der Werkzeugkasten Niederlassung ist eine, durch das Forum Weiterbildung gepflegte, Seminarreihe rund um das Thema Niederlassung.

Der Erste Hilfe Kasten ist ein 12 Module enthaltendes Onlineangebot, zur Vermittlung von Inhalten rund um das Thema Niederlassung, entwickelt im Kompetenzzentrum Weiterbildung Allgemeinmedizin Bayern.

## Zusätzliches Material

Zusätzliches Material Zusätzliches Material
