# Supplementary material for: Setting up of practices by general practitioners in Bavaria with a special focus on bureaucratic processes
Source: Gesundheitswesen. 2025 Jul 18;88(2):113–8. [Article in German] doi: 10.1055/a-2637-3305 (PMC12885622; doi:10.1055/a-2637-3305)
Supplement: Supplementary file 1 — Zusätzliches Material [file 10-1055-a-2637-3305-gesu-2024-09-2144-oa.pdf]

Online-Appendix 1: Beschreibung der Hauptkategorien der Codierung des Projekts „Niederlassungsprozesse von Hausärztinnen und Hausärzten in Bayern mit besonderem Fokus auf bürokratische Vorgänge“

Hauptcode	Beschreibung	Beispiel
Bürokratie im Niederlassungsprozess	In dieser Kategorie wurden alle Faktoren eingeschlossen, welche bürokratische Prozesse im Prozess der Niederlassung beschrieben.	„Also ich fand sehr bürokratisch als ich mich auf diesen Sitz niedergelassen habe, dass ich diese kompletten Anträge, das waren glaube ich einen halben Tag war ich mit den kompletten Anträgen beschäftigt, wo ich sämtliche Nachweise wieder erneut einreichen musste die ich eigentlich schon gefühlt zehn Mal eingereicht hatte erneut meine Approbation von polizeilichem Führungszeugnis über was nicht noch allem und in jedem Antrag wieder genau das gleiche also es war irgendwie Schreibarbeit von, wahrscheinlich ist es übertreiben, ein halber Vormittag aber es war sehr sehr viel und auch rauszufinden welche Anträge ich jetzt für was brauch habe ich als extrem bürokratisch empfunden. Mein Kenntnisstand ist jetzt, dass ich die ganzen Anträge die ich auf den angestellten Kassensitz ausgefüllt habe, dass ich die übernehmen darf, wenn ich mich selber niederlasse aber ich bin mir noch nicht sicher ob das so der Fall sein wird.“
Bürokratie im Praxisalltag	In dieser Kategorie wurden alle Faktoren eingeschlossen, welche bürokratische Prozesse im Praxisalltag beschrieben.	„[…] ich arbeite es irgendwann ab, wenn ich Zeit habe. >Lacht< Also das ist halt, es gehört mit dazu. so einen Schwerbehindertenausweis und die wollen halt einfach viel wissen, die wollen halt wirklich über die letzten zwei Jahre eine Krankheitsgeschichte und das braucht natürlich Zeit bis ich das alles zusammengestellt habe. Klar, reicht es, wenn ich meine Akte aufmache und das mir im Copy Paste und dann habe ich es drüben, aber die wollen halt dann noch detaillierte Untersuchungen und all so einen Kram und das ist halt wahnsinnig aufwendig. Zum einen muss ich die Patienten mir wieder einbestellen und dann muss ich es zu Papier bring und das ist halt das was dann halt Zeit frisst […]“

Unterstützung mit bürokratischen Prozessen	In dieser Kategorie wurden alle Faktoren eingeschlossen, welche die Befragten als unterstützend mit bürokratischen Prozessen in allen Phasen wahrnahmen.	„Nur das was ich schon gesagt habe, also sich möglichst eher breit aufzustellen als ja Einzelpraxis wäre für mich nie in Frage gekommen und verstehe ich auch in der heutigen Zeit nichtmehr dass sich eben wegen dem Ganzen drumherum aber auch der Medizin im Speziellen dass man sich alleine niederlässt. Grade es hören im Umkreis von uns wirklich sehr sehr viele Praxen auf und dementsprechend, vor kurzem hat wieder einer als Einzelpraxis angefangen, das mag zwar passend sein für denjenigen, aber ich hätte es mir nicht vorstellen können, darum ist immer meine Empfehlung, sucht euch mehrere, verteilt es auf mehrere Schultern, dann ist es für jeden ein kleiner, kleiner Teil seines täglichen Werkens sich noch um irgendwas speziell zu kümmern und nicht alles alleine machen zu müssen.“
Veränderung im Zeitverlauf	In dieser Kategorie wurden alle Textstellen eingeschlossen, in welchen die Befragten eine Veränderung ihrer Wahrnehmung im Verlauf der Zeit beschrieben	„Dadurch dass ich es gewöhnt war bei jedem Stellenwechsel schon gefühlt 100 Seiten Papier auszufüllen, eine erneute Befreiung von der Rentenversicherungspflicht, alle Anträge bei der jeweiligen KV für alle Abrechnungsgenehmigungen erneut zu stellen, war das eine geringe Übung das für die Niederlassung nochmal zu machen. Also der Aufwand war nicht viel mehr als im Stellenwechsel in der Anstellung.“
